# Mitochondrial Targeting and pH-Responsive Nanogels for Co-Delivery of Lonidamine and Paclitaxel to Conquer Drug Resistance

**DOI:** 10.3389/fbioe.2021.787320

**Published:** 2021-11-29

**Authors:** Enping Chen, Ting Wang, Junmei Zhang, Xiang Zhou, Yafan Niu, Fu Liu, Yinan Zhong, Dechun Huang, Wei Chen

**Affiliations:** ^1^ Department of Pharmaceutical Engineering, School of Engineering, China Pharmaceutical University, Nanjing, China; ^2^ Engineering Research Center for Smart Pharmaceutical Manufacturing Technologies, Ministry of Education, School of Engineering, China Pharmaceutical University, Nanjing, China

**Keywords:** multidrug resistance reversal, mitochondrial targeting, pH responsive, nanogel, combination therapy

## Abstract

Multidrug resistance (MDR) is one of the leading causes of the failure of cancer chemotherapy and mainly attributed to the overexpression of drug efflux transporters in cancer cells, which is dependent on adenosine triphosphate (ATP). To overcome this phenomenon, herein, a mitochondrial-directed pH-sensitive polyvinyl alcohol (PVA) nanogel incorporating the hexokinase inhibitor lonidamine (LND) and the chemotherapeutic drug paclitaxel (PTX) was developed to restore the activity of PTX and synergistically treat drug-resistant tumors. The introduction of 2-dimethylaminoethanethiol (DMA) moiety into the nanogels not only promoted the drug loading capacity but also enabled the lysosomal escape of the nanogels. The subsequent mitochondrial targeting facilitated the accumulation and acid-triggered payload release in the mitochondria. The released LND can destroy the mitochondria by exhausting the mitochondrial membrane potential (MMP), generating reactive oxygen species (ROS) and restraining the energy supply, resulting in apoptosis and susceptibility of the MCF-7/MDR cells to PTX. Hence, the nanogel-enabled combination regimen of LND and PTX showed a boosted anti-tumor efficacy in MCF-7/MDR cells. These mitochondrial-directed pH-sensitive PVA nanogels incorporating both PTX and LND represent a new nanoplatform for MDR reversal and enhanced therapeutic efficacy.

## Introduction

Chemotherapy remains one of the major means for treating tumors; however, the therapeutic efficacy is perplexed by many reasons including the multidrug resistance (MDR) (Lage 2008; Holohan et al., 2013; Rebucci and Michiels, 2013). The underlying mechanisms are complex and can be mainly summed up in the increased drug efflux from the cytoplasm to the extracellular compartment *via* the adenosine triphosphate (ATP)-binding cassette (ABC) transporters in the cells such as P-glycoprotein (P-gp) and multidrug resistance-associated protein 1 (MRP1) driven by the energy supply of ATP (Cole 2014; Kartal-Yandim et al., 2016; Assaraf et al., 2019). In order to reverse MDR, many strategies have been dedicated to the MDR inhibition by interfering the energy supply, re-sensitizing the tumor cells to the chemotherapeutic drugs (Abdallah et al., 2015; Assanhou et al., 2015; Li et al., 2016; Tu et al., 2018; Gao et al., 2019; Cheng et al., 2021; Liu et al., 2021).

Lonidamine (LND), which is a hexokinase inhibitor, blocks the energy supply and mitochondrial respiration, leading to mitochondrial dysfunction (Floridi et al., 1981; Price et al., 1996; Nath et al., 2016). In addition, LND triggers cell apoptosis by dissipating mitochondrial membrane potential (MMP) and generating reactive oxygen species (ROS). The combination of LND as a chemosensitizer with chemotherapeutic drugs provides an effective strategy for treating drug-resistant tumors (Li et al., 2002; Nath et al., 2013; Nath et al., 2015; Huang et al., 2020). Nevertheless, the poor water solubility and the lack of targetability of the drugs compromised their efficacy and limited their applications. Nanomedicine by virtue of its unique properties, such as the solubilization of the hydrophobic drugs and the delivery of the payload to the tumor sites *via* the enhanced permeability and retention (EPR) effect, has received widespread attention (Golombek et al., 2018; Majumder et al., 2019; Cao et al., 2020; Qiao et al., 2021). Nowadays, multifunctional nanomedicine integrating various modules of targeting (Qiao et al., 2019), lysosomal escaping, stimuli sensitivity, etc. (Pu et al., 2019; Zong et al., 2020; Zhong et al., 2021; Zong et al., 2021) could further improve the drug delivery efficacy, maximizing the therapeutic outcomes (Chen et al., 2017; Shahriari et al., 2019; Yang et al., 2019; He et al., 2021; Peng et al., 2021). It should be noted that the mitochondrial-targeting strategy plays an essential role in fully taking advantage of LND (Li et al., 2013; Zhang et al., 2015; Liu et al., 2017; Guo et al., 2021). Additionally, the biosafety of nanomedicine is a big concern.

**SCHEME 1:**
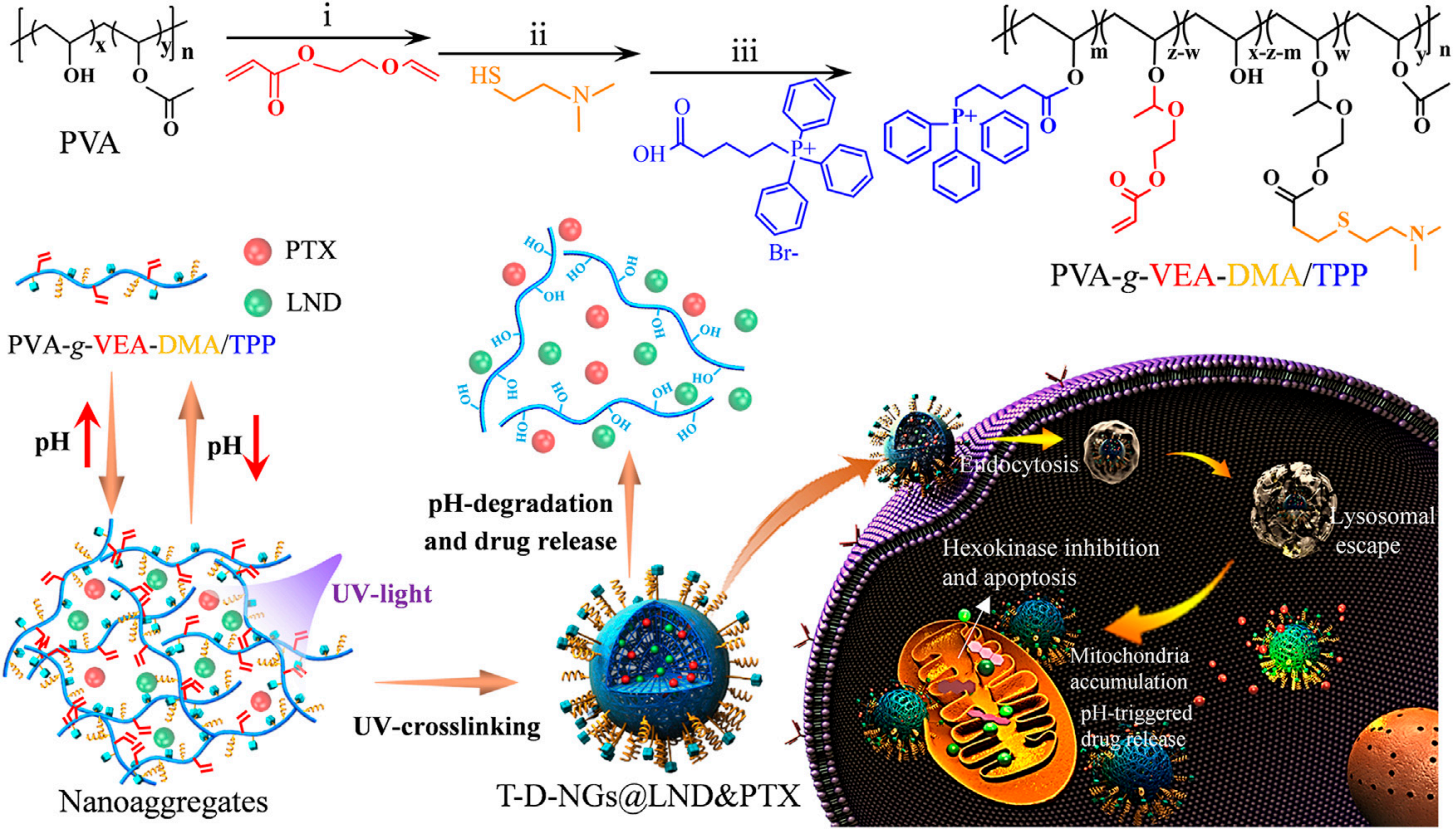
Schematic illustrations for the constituent, fabrication, and destruction of T-D-NGs@LND and PTX and its endocytosis, lysosomal escape, mitochondrial orientation, and acid-triggered drug release within drug-resistant tumor cells for synergistic effect including LND-induced apoptosis, drug resistance alleviation, and restored chemotherapeutics of PTX.

In this study, we have constructed a triphenylphosphine (TPP)-installed pH-sensitive biocompatible polyvinyl alcohol (PVA) nanogel containing 2-dimethylaminoethanethiol (DMA) moiety for mitochondrial-directed co-delivery of LND and the chemotherapeutic drug paclitaxel (PTX) in drug-resistant tumors. The nanogels (T-D-NGs@LND and PTX) were fabricated by *in situ* crosslinking of the acrylate in PVA derivative under UV light and the concurrent co-loading of LND and PTX *via* hydrophobic and/or electrostatic interactions. After the internalization in the drug-resistant tumor cells, T-D-NGs@LND and PTX escaped from the lysosome due to the proton sponge effect of DMA moiety, effectively accumulated in the mitochondria with the orientation of TPP module, and rapidly released the payload resulting from the acid-triggered cleavage of the acetal linker within the network of nanogels (Scheme 1). The unleashed LND acted on the mitochondria and de-energized the cancer cells, resulting in cell apoptosis and making the cells more susceptible to the released PTX. The nanogel-mediated drug resistance alleviation and synergistic effect significantly boosted the anti-tumor activity in MCF-7/MDR cells, holding a great potential for conquering MDR.

## Experimental Section

### Preparation of DMA-Modified Vinyl Ether Acrylate-Functionalized PVA

Vinyl ether acrylate (VEA) and VEA-functionalized PVA (PVA-*g*-VEA) were prepared based on the previous reports (Chen et al., 2017). For the synthesis of DMA-functionalized PVA-*g*-VEA (PVA-*g*-VEA-DMA), DMA and Et_3_N (20 μl) were added to a 10-ml solution of PVA-*g*-VEA (0.413 g, 0.024 mmol) in methanol under stirring, and the reaction was continued for 12 h. Afterwards, the above mixture solution was condensed by rotary evaporation. Finally, PVA-*g*-VEA-DMA was purified using the precipitant of ice-cold diethyl ether and then dried using the vacuum drier.

### Preparation of TPP-Functionalized PVA-*g*-VEA-DMA

To synthesize TPP-modified PVA-*g*-VEA-DMA, a mixture of TPP (70.5 mg, 0.16 mmol), DCC (49.1 mg, 0.24 mmol), and DMAP (20.3 mg, 0.17 mmol) was dissolved in dimethyl sulfoxide (DMSO) (10 ml), and the obtained solution was vigorously stirred for 1 h for activation of the carboxyl group in TPP. Subsequently, a solution of PVA*-g-*VEA-DMA (100 mg) in DMSO (5 ml) was slowly added for a 24-h reaction under stirring. After that, PVA-*g*-VEA-DMA/TPP was purified *via* extensive dialysis [molecular weight cut off (MWCO): 3.5 kDa] against methanol for 24 h, concentrated through rotary evaporation, precipitated in ice-cold diethyl ether, as well as vacuum dried.

### Preparation and Characterization of Nanogels

Acid-responsive nanogels were prepared using the UV-crosslinking method. Briefly, the nanogels were obtained by dissolving the polymer of PVA-*g*-VEA-DMA/TPP in water (1 mg/ml) with the addition of a photoinitiator (I2959, 5 wt% of polymer), adjusting solution pH to 6.8, and stirring under UV exposure for 10 min. To prepare drug-loaded nanogels, a solution of PTX and LND in DMSO (10 mg/ml) was added in the aqueous polymer solution with a feeding ratio of 10%. Through adjusting the solution pH to 6.8 and *in situ* crosslinking of acrylate in the polymers under UV light for 10 min, PTX and LND co-loaded nanogels were obtained. A Millipore ultrafiltration centrifugal tube with a MWCO of 10,000 was used to remove the free drugs.

### pH-Induced Degradation of Nanogels and *In Vitro* Release Profile of LND and PTX

pH-induced degradation of nanogels was studied by detecting the nanogel size change *via* dynamic light scattering (DLS) in different conditions (pH 7.4 and 5.0) for different periods (0, 6, and 24 h). The unleash behavior of LND and PTX-loaded nanogels was explored in different conditions (pH 7.4 and 5.0) through the dialysis procedure. In brief, a dialysis bag (MWCO: 3,500) containing 1 ml of PTX and LND co-loaded nanogel suspension was placed into a large-volume tube with 20 ml of the corresponding medium under a continuous shaking at 100 rpm at 37°C. At various time intervals (0, 2, 4, 6, 8, 10, 24, 52, 56, and 72 h), 5 ml of the medium in the tube of each group was extracted and supplemented by the newly prepared medium. The collected media of each group were concentrated and subjected to HPLC for quantification of the released PTX and LND.

### Lysosomal Escape and Co-Localization Into the Mitochondria

MCF-7/MDR cells (1 × 10^5^ cell/well) were seeded into a 24-well plate containing a rounded coverslip in each well. After 24 h, fluorescein isothiocyanate (FITC)-labeled NGs, D-NGs, and T-D-NGs were added and incubated with the cells for 8 h. Then, the medium was removed, and the cells were incubated with 500 μl of serum-free media containing 200 nM MitoTracker® Red CM-H2XRos probes at 37°C for 20 min or 500 μl of serum-free media containing 50 nM LysoTracker® Red DND-99 probes at 37°C for 30 min. After washing thrice in phosphate-buffered saline (PBS) and fixation of the cells using 4% paraformaldehyde for 20 min, 4′,6-diamidino-2-phenylindole (DAPI) was added for 10-min staining of the cell nucleus, and the excess DAPI was removed by washing thrice in PBS. Finally, the fluorescence images of each group were obtained by confocal laser microscope (CLSM, LSM700, Zeiss, Germany) and processed using the ZEN imaging software.

### Mitochondrial Membrane Potential (Δψm) Depolarization

To measure the mitochondrial depolarization, the cationic fluorochrome JC-1 was employed to detect mitochondrial membrane potential change. Briefly, MCF-7/MDR cells were seeded in a 12-well plate (1 × 10^5^ cells/well) and incubated for 24 h. Then, free LND, NGs@LND, D-NGs@LND, and T-D-NGs@LND at a LND dosage of 50 μg/ml were severally added and incubated with the cells for 12 h. Thereafter, the cells were washed with PBS followed by incubation with 0.0125 M JC-1 at 37°C for 20 min. After washing thrice in PBS, the cells were observed using a fluorescence microscope (IX73, Olympus, Japan).

### ROS Level Detection

A ROS assay kit (Beyotime, China) was utilized for the detection of the intracellular ROS level. Briefly, MCF-7/MDR cells were cultured in a 12-well plate (1 × 10^5^ cells/well) and incubated for 24 h. Then, free LND, NGs@LND, D-NGs@LND, and T-D-NGs@LND at a LND dosage of 50 μg/ml were respectively added and incubated with the cells for 12 h. Afterwards, the cells were washed with PBS for two times and stained with dichlorodihydro-fluorescein diacetate (DCFH-DA) for 20 min. After washing thrice in PBS and fixation of the cells using 4% paraformaldehyde for 20 min, DAPI was added for a 10-min staining of the cell nucleus, and the excess DAPI was removed by washing thrice in PBS. Finally, the cells were observed using a fluorescence microscope (IX73, Olympus, Japan).

### ATP Content Detection

An ATP assay kit (Beyotime, China) was utilized for the detection of the intracellular ATP levels. MCF-7/MDR cells were cultured in a six-well plate (1 × 10^5^ cell/well) for 24 h. After that, free LND, NGs@LND, D-NGs@LND, and T-D-NGs@LND at a LND dosage of 50 μg/ml were respectively added and incubated with the cells for 12 h. The intracellular ATP level was measured according to the manufacturer’s protocol.

### *In Vitro* Anti-Tumor Activity

To evaluate the apoptosis-inducing effect of different LND formulations, MCF-7/MDR cells were seeded into 12-well plates (1 × 10^5^ cell/well) and cultured for 24 h at 37°C. Then, different LND formulations (50 μg/ml LND) were added and incubated with the cells for 12 h. After that, the cells were digested and collected for Annexin V-FITC/propidium iodide (PI) staining based on the manufacturer’s instructions and analyzed by flow cytometry.

MTT assay was utilized to study the cytotoxicity of MCF-7/MDR cells receiving various formulations. The cells were cultured in 96-well plates for 24 h and then incubated with various formulations or free drug. After 48-h incubation, MTT solution was added for 4-h incubation. Then, the culture medium was replaced by DMSO for detection under a microplate reader with the absorbance at 490 nm.

### Statistical Analysis

Data were expressed as mean ± standard deviation. Differences between groups were assessed by a two-tailed unpaired Student’s *t*-test or one-way ANOVA with Tukey’s *post hoc* test. The level of significance was set at probabilities of *p < 0.05, **p < 0.01, and ***p < 0.001.

## Results and Discussion

### Synthesis and Characterization of TPP-Modified PVA Nanogels

**FIGURE 1:**
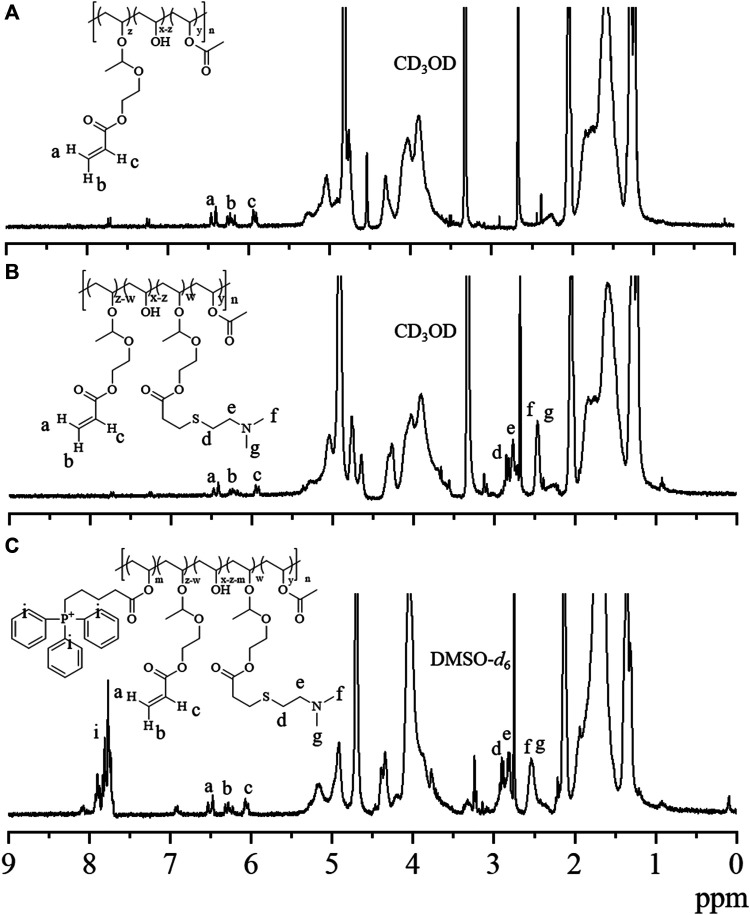
^1^H NMR spectra of **(A)** PVA-*g*-VEA (300 MHz, CD_3_OD), **(B)** PVA-*g*-VEA-DMA (300 MHz, CD_3_OD), and **(C)** PVA-*g*-VEA-DMA/TPP (300 MHz, DMSO-*d*_6_). VEA, vinyl ether acrylate; PVA-*g*-VEA, VEA-functionalized PVA; PVA-*g*-VEA-DMA, DMA-functionalized PVA-*g*-VEA; TPP, triphenylphosphine.

We started with the synthesis of PVA-*g*-VEA/VEA-DMA/TPP polymer, in which the pendent VEA, positively charged DMA, and mitochondria-recognizable TPP were successively conjugated onto the water-soluble PVA backbone (Scheme 1). The VEA was firstly grafted onto PVA polymers *via* an acetalization reaction between vinyl ether and hydroxyl group in the presence of PTSA. The signals at *δ* 6.6 and *δ* 5.85–6.45 attributable to the vinyl protons of VEA and newly formed acetal methine proton, respectively, revealed the successful conjugation of VEA to the polymers, and the VEA grafting ratio was determined to be about 6.1% by comparing the integral of the peaks at *δ* 1.3 and at *δ* 6.6 (Figure 1A). DMA was further conjugated with acrylate in PVA-VEA, yielding a DMA functionality of about 2.66% with 3.44% acrylate left for UV-mediated crosslinking according to the ^1^H NMR spectra (Figure 1B). Lastly, the obtained polymeric conjugates were coupled with TPP on the backbone using DCC and DMAP *via* esterification, and the content of the TPP moiety on PVA-VEA-DMA-TPP was about 1.3 wt% as revealed from the integrals of the peaks at *δ* 7.68 of benzene protons and at *δ* 1.3 (Figure 1C).

**FIGURE 2:**
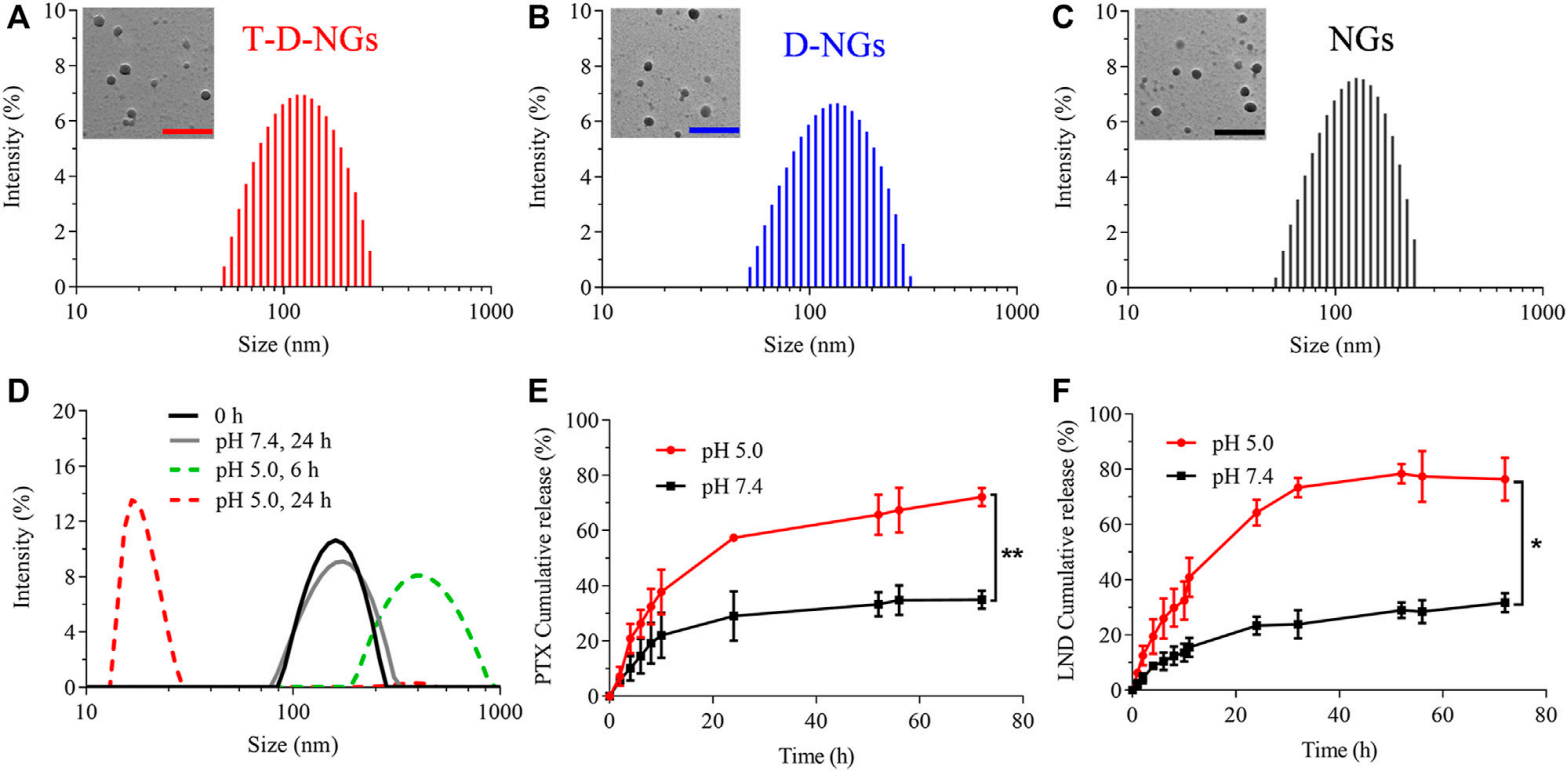
Size distribution of **(A)** T-D-NGs, **(B)** D-NGs, and **(C)** NGs determined by DLS and TEM (inset). Scale bars: 500 nm. **(D)** pH-triggered size variation of T-D-NGs for different times (0, 6, and 24 h) determined by DLS. *In vitro* **(E)** PTX and **(F)** LND release from T-D-NGs (2 mg/ml) at 37°C (*n* = 3, *p < 0.05, **p < 0.01). DLS, dynamic light scattering; TEM, transmission electron microscopy; PTX, paclitaxel; LND, lonidamine.

T-D-NGs were then obtained by dissolving the polymer of PVA-*g*-VEA/VEA-DMA/TPP in water, adjusting pH to 7.8, crosslinking acrylate in the presence of I2959 under UV light. D-NGs and NGs were prepared in a similar way. As displayed in Figure 2A–C, T-D-NGs, D-NGs, and NGs presented a typical spherical shape with uniform sizes of about 170 nm. pH-responsive T-D-NGs were explored by detecting the size change of T-D-NGs in acetate buffer (pH 5.0) for different times *via* DLS measurement. T-D-NGs displayed a swelled structure with a size of about several hundred nanometers after 6 h and later a small-sized particle with a size of about 17 nm after 24 h, whereas the size of T-D-NGs maintained almost the same at pH 7.4 after 24-h incubation (Figure 2D). These results revealed that T-D-NGs possessed a good colloidal stability in the physiological condition while suffered from the destruction at pH 5.0 that mimicked intracellular acidic condition. It should be noted that the swelled structure of T-D-NGs in response to acidic condition in their initial stage was ascribed to their partial cleavage of the acetal linkers in the network of T-D-NGs, which ultimately led to complete disassociation and small-sized conjugates in the acidic condition.

### Preparation and Characterization of Drug-Loaded Nanogels

LND, which is a small-molecule hexokinase inhibitor, and chemotherapeutic PTX were co-loaded into the T-D-NGs *via* hydrophobic and/or electrostatic interactions. LND and PTX-loaded nanogels showed increased sizes in comparison to the blank nanogels as determined by DLS (Table 1). The introduction of DMA and TPP moieties in the nanogels dramatically improved drug loading content (DLC) from 2 wt% to about 5 wt% at a theoretical DLC of 10 wt%, which was probably due to their increased hydrophobicity and electropositivity.

**TABLE 1:** Characteristics of different drug-loaded nanogels^a^

Nanogels	DLE (%)^b^		DLC (%)^b^		Size^c^	PDI^c^
	PTX	LND	PTX	LND		
NGs	23.01 ± 1.0	20.74 ± 0.2	2.25 ± 0.1	2.03 ± 0.1	165.6 ± 0.6	0.22
D-NGs	46.70 ± 5.5	57.69 ± 6.7	4.46 ± 0.5	5.45 ± 0.6	211.7 ± 3.2	0.15
T-D-NG	53.21 ± 0.9	74.92 ± 0.4	5.05 ± 0.1	5.05 ± 0.1	182.3 ± 6.1	0.21

a The concentration of nanogels was set at 1.0 mg/ml.

b Determined by HPLC.

c Determined by DLS.

The *in vitro* LND and PTX release profiles from T-D-NGs were studied at various pH, i.e., pH 5.0 and pH 7.4. As displayed in Figure 2E, F, a small amount of LND (31.7%) and PTX (34.5%) was leaked from T-D-NGs at pH 7.4 after 72 h, respectively, whereas more than 70% of LND and PTX were released at pH 5.0 after 72 h, further confirming pH-triggered disassociation of the nanogels and the subsequent payload release.

### Escape From Lysosomes and Mitochondrial Targeting

**FIGURE 3:**
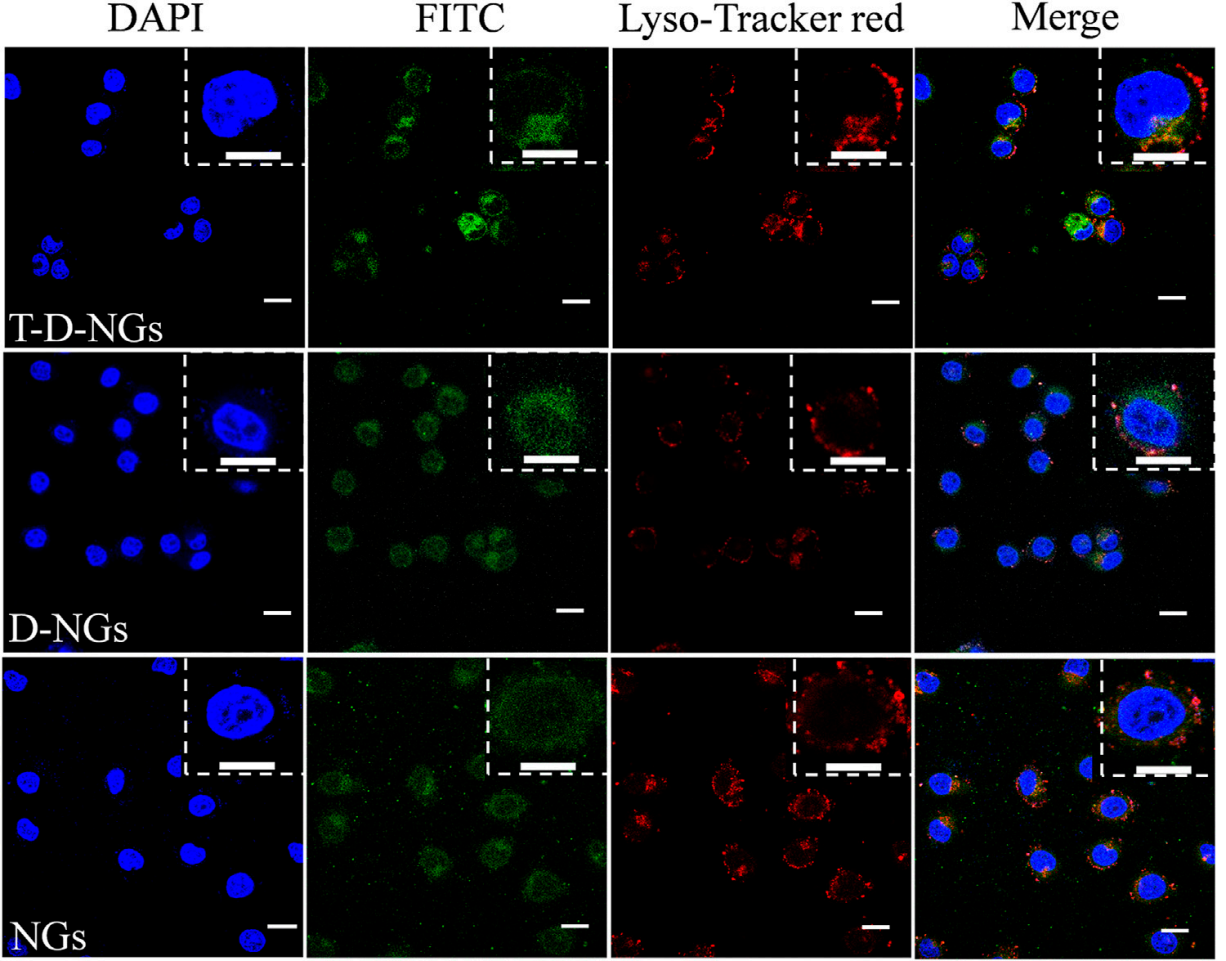
CLSM images of MCF-7/MDR cells treated with different FITC-labeled nanogels (500 μg/ml) following 8-h incubation. The lysosomes were stained with LysoTracker Red, and the cell nuclei were stained with DAPI (blue signal). Scale bar: 20 μm. CLSM, confocal laser microscope; FITC, fluorescein isothiocyanate; DAPI, 4′,6-diamidino-2-phenylindole.

**FIGURE 4:**
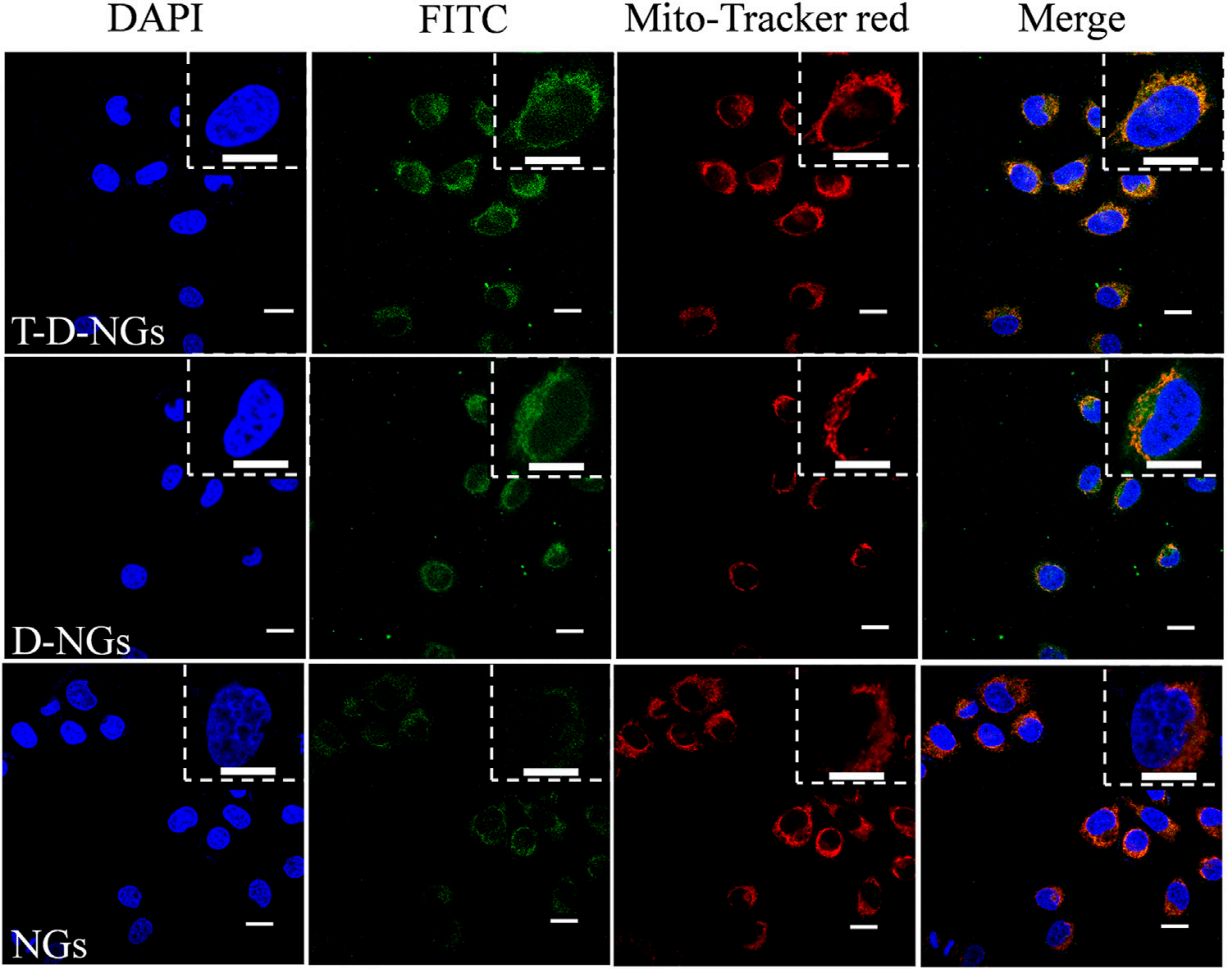
CLSM images of MCF-7/MDR cells treated with different FITC-labeled nanogels (500 μg/ml) following 12-h incubation. The mitochondria were stained with MitoTracker Red, and the cell nuclei were stained with DAPI (blue signal). Scale bars: 20 μm.

The cellular behaviors of T-D-NGs were investigated by using CLSM in MCF-7/MDR cells. T-D-NGs, D-NGs, and NGs were labeled with FITC for CLSM imaging, and the results showed that FITC-labeled NGs (FITC-NGs, green signal) were captured by lysosomes (red signal) with colocalized orange signal within cells for 8-h incubation, while most of the green signal of FITC-labeled T-D-NGs (FITC-T-D-NGs) and FITC-labeled D-NGs (FITC-D-NGs) were separated from the red signal of lysosomes after 8 h, indicating the successful escape of FITC-T-D-NGs and FITC-D-NGs from lysosomes with the aid of DMA module in the nanogels (Figure 3). In the following, we evaluated the mitochondrial-targeting ability of FITC-T-D-NGs, FITC-D-NGs, and FITC-NGs by staining the mitochondria with MitoTracker (red). After 12 h, a large area of FITC-T-D-NGs were overlapped with the mitochondria, resulting in intense orange fluorescence in the merged image (Figure 4). In contrast, FITC-D-NGs presented separated green and red fluorescence, and hardly any green signal was observed in the FITC-NG group with only red fluorescence in the merged image. All the above results demonstrated that FITC-T-D-NGs could efficiently escape from the lysosomes and target the mitochondria for facilitating LND delivery, while FITC-NGs and FITC-D-NGs either got trapped in the lysosomes or lacked mitochondrial orientation.

### Dysfunction of the Mitochondria

**FIGURE 5:**
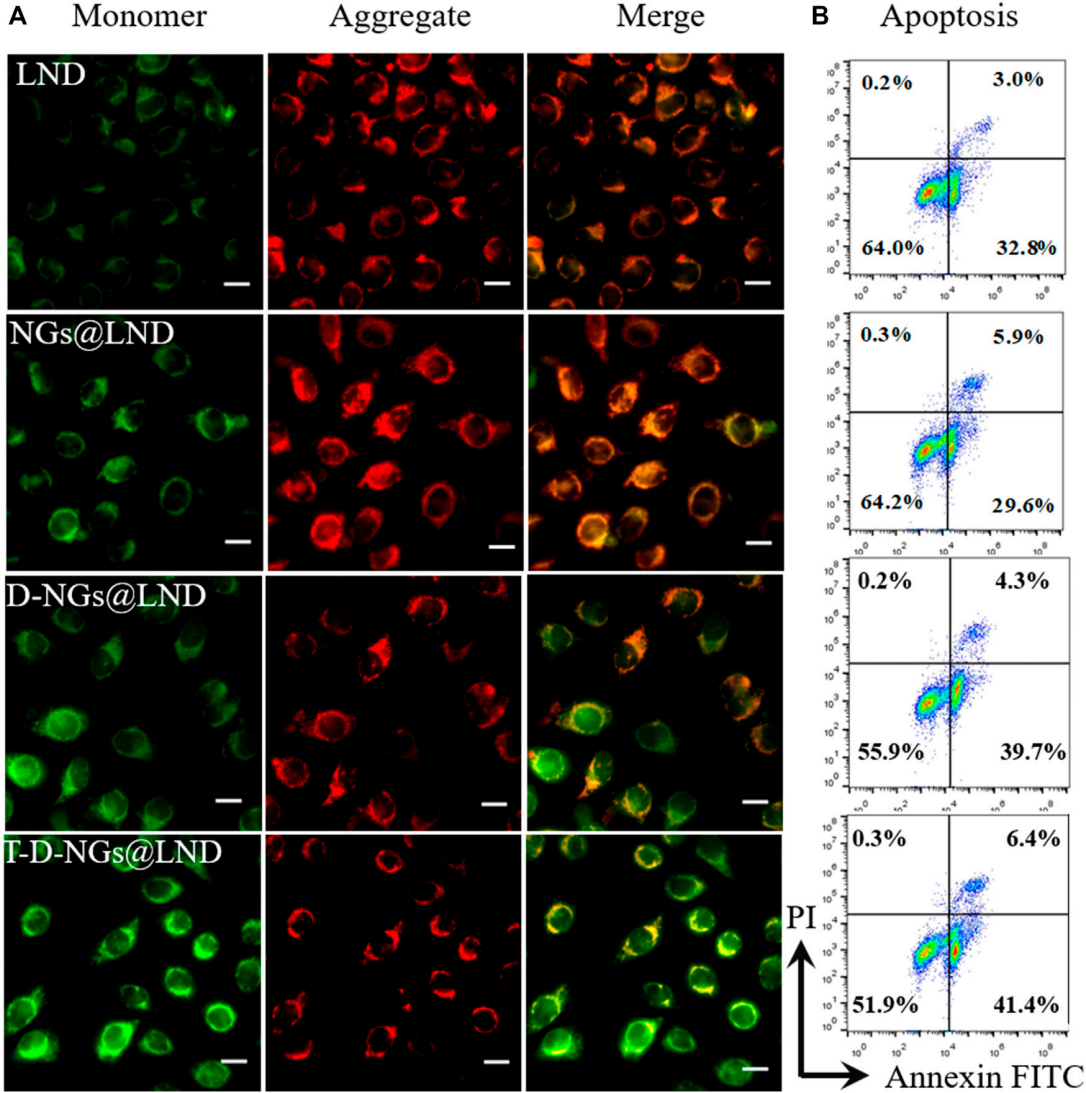
**(A)** The MMP depolarization level of MCF-7/MDR cells incubated with LND, NGs@LND, D-NGs@LND, and T-D-NGs@LND for 12 h. The mitochondrial channel was stained with JC-1. Scale bars: 20 μm. **(B)** Cell apoptosis studies with treatments of various LND-loaded nanogels for 12 h. MMP, mitochondrial membrane potential.

To explore the underlying mechanism of LND-loaded T-D-NGs (T-D-NGs@LND) in interfering mitochondrial functions, the measurements of MMP, ROS production, and ATP secretion in MCF-7/MDR cells were performed. JC-1 staining was firstly employed, which served as a fluorescence probe to detect MMP. JC-1 forms aggregates with red fluorescence at a high MMP, while remains monomers with green fluorescence at a low potential level, which is indicative of early apoptosis. After 12-h incubation, free LND displayed minimal green signal and intense red signal level comparable to the PBS group in MCF-7/MDR cells due to its slow diffusion and poor mitochondrial targeting (Figure 5A; Supplementary Figure S1). NGs@LND slightly increased the green fluorescence to some extent and maintained the strong red fluorescence, revealing an insufficient dissipation of MMP because of being trapped in lysosomes and limited accumulation in the mitochondria as demonstrated above. D-NGs@LND ameliorated this phenomena because of its lysosomal escape aided by DMA moieties in the nanogels. T-D-NGs@LND was the most effective in dissipating MMP with the strongest green fluorescence concomitant with the weakest red fluorescence, which was ascribed to its lysosomal escape, mitochondrial accumulation, and effective LND release triggered by acid within cells.

**FIGURE 6:**
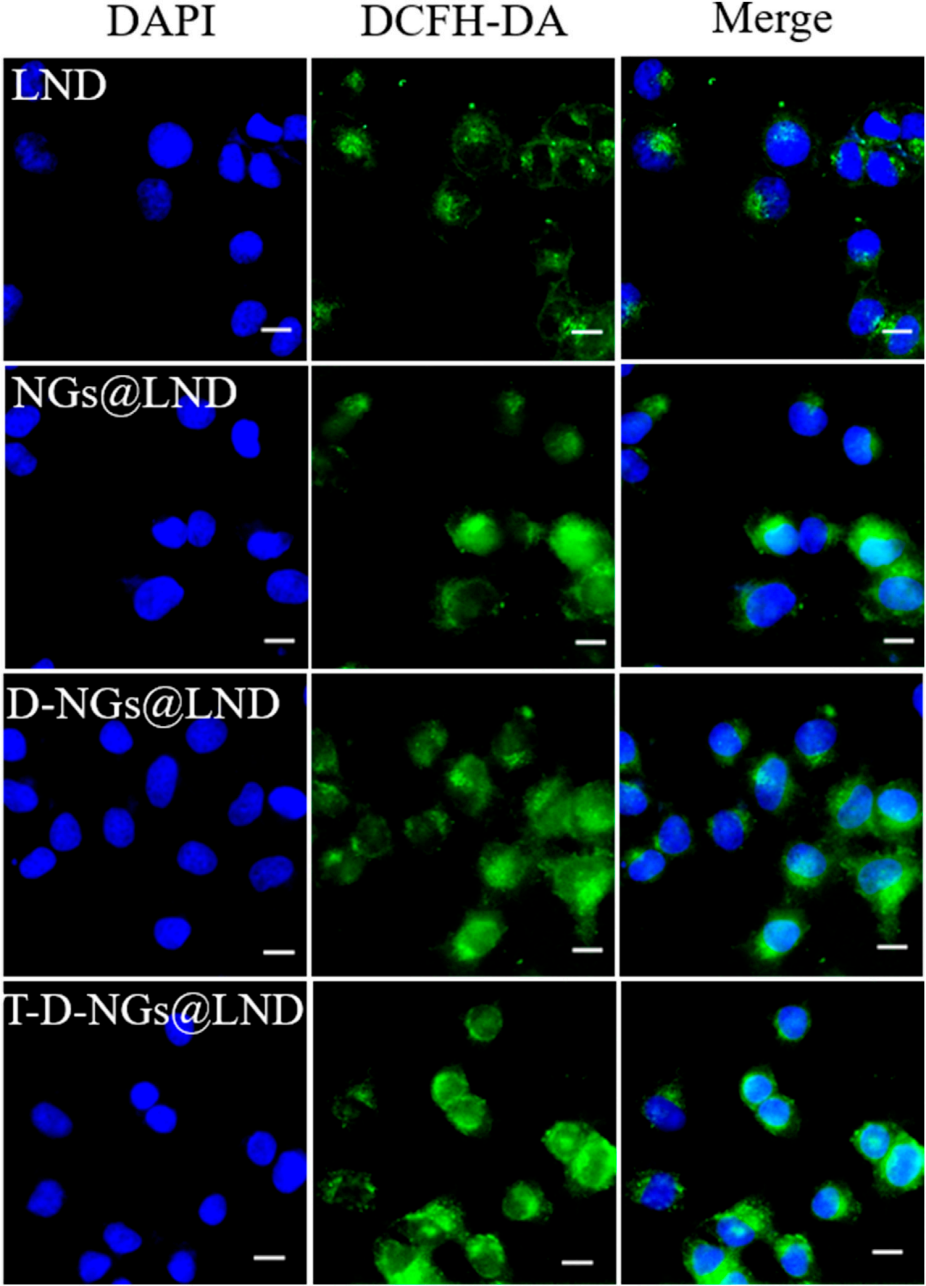
Intracellular ROS level in MCF-7/MDR cells receiving LND, NGs@LND, D-NGs@LND, and T-D-NGs@LND for 12 h using a probe of DCFH-DA. Scale bars: 20 μm. ROS, reactive oxygen species; DCFH-DA, dichlorodihydro-fluorescein diacetate.

ROS production causes oxidative cell damage at a high level, which was another mechanism involved in cancer cell apoptosis *via* the mitochondrial pathway. ROS production level was detected by a fluorescence probe of DCFH-DA *via* CLSM imaging. The trend of the intracellular ROS production level was similar with the results of the dissipation of MMP (Figure 6; Supplementary Figure S2), verifying that T-D-NGs@LND induced a large amount of intracellular ROS production in the mitochondria, potentially for anti-tumor applications.

Cumulative evidences demonstrated that LND had the potential to inhibit hexokinase enzyme that was implicated in ATP generation and mitochondrial respiration. ATP secretion from the MCF-7/MDR cells receiving various LND formulations was estimated through the ATP assay kit. As expected, T-D-NGs@LND significantly decreased ATP secretion from the cells with a decline of about 60.9% versus 43.48%, 30.44%, and 15.95% for D-NGs@LND, NGs@LND, and free LND severally after 12-h incubation (Supplementary Figure S3). Such a pronounced inhibition of ATP secretion in MCF-7/MDR cell induced by T-D-NGs@LND further confirmed the precise and efficient delivery of LND into the mitochondria and also laid a solid foundation for MDR reversal by suppressing P-gp that was dependent on ATP.

### Anti-Tumor Activity

**FIGURE 7:**
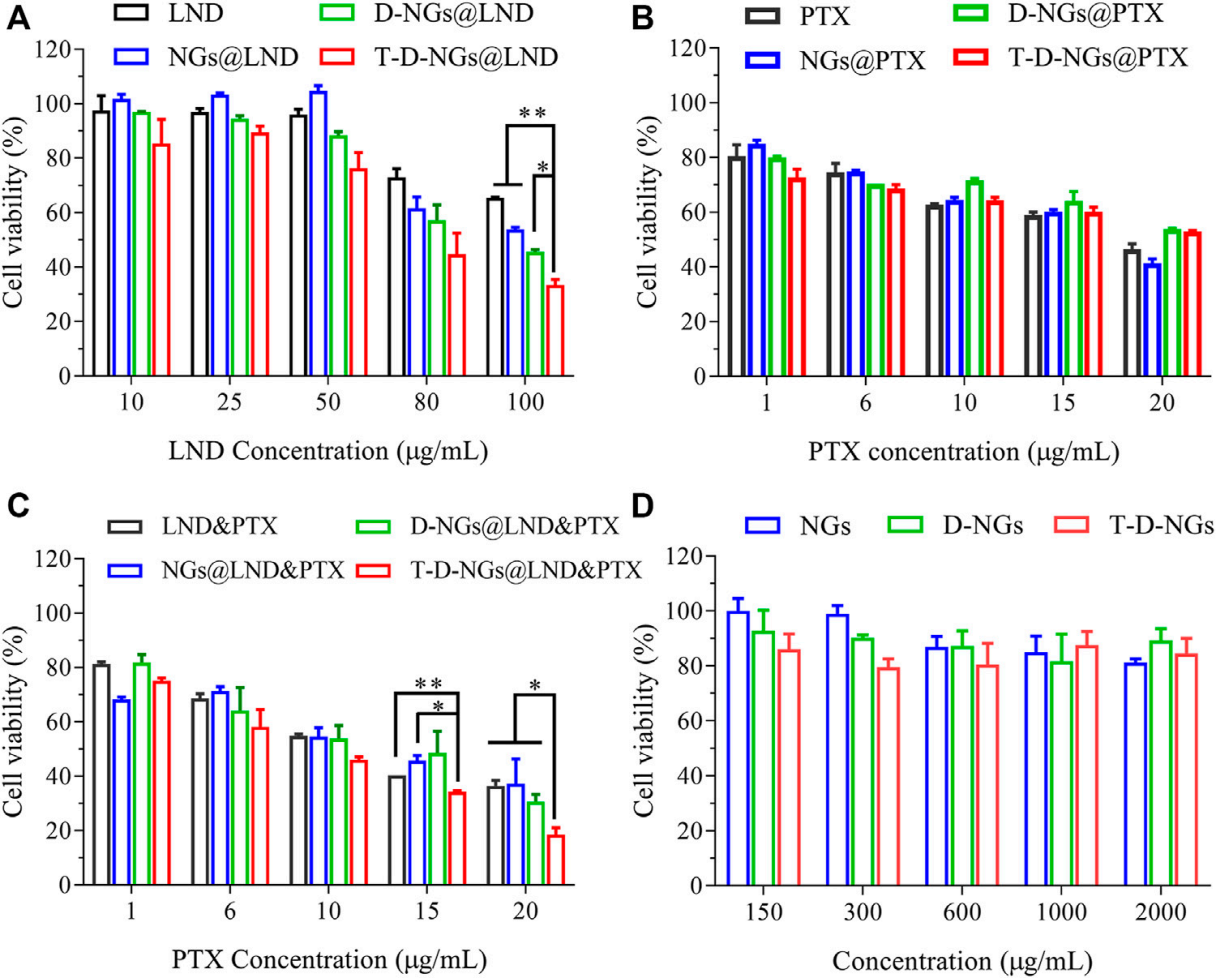
Cytotoxicity of MCF-7/MDR cells after 48-h treatments of **(A)** LND-loaded nanogels, **(B)** PTX-loaded nanogels, **(C)** LND and PTX-loaded nanogels (LND: 50 μg/ml), and **(D)** drug-free nanogels (*n* = 3, *p < 0.05, **p < 0.01).

The cell apoptosis with the treatments of various LND formulations *via* the mitochondrial pathway was explored *via* Annexin V-FITC/PI staining through flow cytometry. LND could induce cell apoptosis *via* several mechanisms like destruction of MMP and oxidative stress of overproduced ROS in the mitochondria as noted above. Total apoptotic rates of about 47.8% was found in T-D-NGs@LND-treated cells, which was higher than the D-NGs@LND (44%), NGs@LND (35.5%), and free LND (35.8%) groups (Figure 5B). Moreover, T-D-NGs@LND caused both higher early (41.4%) and late (6.4%) apoptosis than other treatments. MTT assay was also carried out and showed that all of the LND formulations led to a dose-dependent cytotoxicity toward MCF-7/MDR cells, among which T-D-NGs@LND displayed the most potent anti-tumor activity with a cell viability of about 33.47% at a LND concentration of 100 μg/ml for 48 h (Figure 7A). Taken together, T-D-NGs@LND was demonstrated to exert direct tumor cell-killing ability of LND by overcoming lysosomal trap, navigating into the mitochondria and efficiently releasing LND.

Furthermore, the potential role of LND for drug resistance reversal and chemo-sensitization was utilized to reactivate the treatment ability of PTX in MCF-7/MDR cells. Thus, the combination of LND and PTX in T-D-NGs could bring about a synergistic anti-tumor activity by fully exerting tumor cell killing of both LND and PTX. To verify the above assumption, MTT assay was performed in MCF-7/MDR cells receiving PTX- and/or LND-loaded nanogels for 48 h. As displayed in Figure 7B, all the PTX formulations and free PTX revealed mild cytotoxicity toward the cells with cell viabilities more than 40% even at a high PTX concentration of 20 μg/ml, reflecting drug resistance of the cells and inactivation of PTX formulations. The combination of LND and PTX in various formulations showed cytotoxicity toward the cells in a PTX dose-dependent fashion at a fixed LND concentration of 50 μg/ml (Figure 7C). As predicted, T-D-NGs@LND and PTX remarkably boosted the tumor cell-killing activity with a cell viability of about 18.59% at LND and PTX concentrations of 50 and 20 μg/ml for 48 h, which was much lower than T-D-NG-enabled monotherapy (T-D-NGs@LND and T-D-NGs@PTX), revealing the reversal of drug resistance and the synergistic therapeutic efficacy of unleashed LND and PTX. The half maximal inhibitory PTX concentration (IC_50_) of T-D-NGs@LND and PTX (LND: 50 μg/ml) was also calculated, which was determined to be 7.44 μg/ml. T-D-NGs@LND and PTX also displayed a much higher anti-tumor activity as compared with D-NGs@LND and PTX (IC_50_: 10.70 μg/ml), NGs@LND and PTX (IC_50_: 11.96 μg/ml), and LND and PTX (IC_50_: 11.49 μg/ml), further verifying T-D-NG-mediated lysosomal escape, mitochondrial accumulation, and acid-triggered payload release for fully exerting the efficacy of both drugs. In the meanwhile, the cytotoxicities of drug-free nanogels toward the cells were also assessed, and the results showed cell viabilities of 80%–100% at nanogel concentrations ranging from 0.15 to 2 mg/ml (Figure 7D), confirming the superior biocompatibility of the nanogels, which was mainly made up of FDA-approved biocompatible PVA backbone.

## Conclusion

In conclusion, we have developed mitochondrial-directed pH-sensitive nanogels incorporating LND and PTX (T-D-NGs@LND and PTX) for drug resistance reversal and synergistic therapy against drug-resistant tumors. After internalization into MCF-7/MDR cells, T-D-NGs@LND and PTX efficiently escaped from the lysosomes with the aid of DMA moiety in the nanogels, accumulated into the mitochondria, and rapidly released the payload in the acidic conditions. The released LND, on one hand, directly induced cell apoptosis *via* dampening the mitochondrial membrane potential, promoting oxidative stress from the ROS production; on the other hand, it facilitated drug resistance reversal and sensitization of PTX activity by blocking the energy source and thus inhibiting drug resistance-related P-gp expression. As a result, T-D-NGs@LND and PTX demonstrated a significantly potent anti-tumor activity against MCF-7/MDR cells with an extremely low IC_50_ of 7.44 μg/ml PTX (LND: 50 μg/ml) after 48-h treatment and a synergistic effect of LND and PTX. Our work provides a promising and synergistic strategy to conquer tumor MDR.

## Data Availability

The original contributions presented in the study are included in the article/Supplementary Material; further inquiries can be directed to the corresponding authors.
