# Supplementary material for: Mitochondrial Targeting and pH-Responsive Nanogels for Co-Delivery of Lonidamine and Paclitaxel to Conquer Drug Resistance
Source: Front Bioeng Biotechnol. 2021 Nov 29;9:787320. doi: 10.3389/fbioe.2021.787320 (PMC8667579; doi:10.3389/fbioe.2021.787320)
Supplement: Supplementary file 1 [file DataSheet1.docx]

Supplementary Material

# Materials

Ethylene glycol vinyl ether (98%), acryloyl chloride (98%), 2-hydroxy-4’-(2-hydroxyethoxy)-2-methylpropiophenone (I2959, 98%), (4-carboxybutyl) triphenylphosphonium bromide (TPP, 98%), 2-dimethylaminoethanethiol hydrochloride (DMA), dicyclohexylcarbodiimide (DCC, 98%), 4-dimethylaminopyridine (DMAP, 97%), triethylamine (Et_3_N, 99%), paclitaxel (PTX, 99%), *p*-toluenesulfonic acid monohydrate (PTSA, 98%) and polyvinyl alcohol (PVA, 87.0–89.0% hydrolyzed, *M*_w_ = 15,000 g/mol) were purchased from Energy Chemical (Shanghai, China). Lonidamine (LND) was acquired from Zhongshuo Co. Ltd. (Beijing, China).

# Characterization

^1^H NMR spectra were recorded on a Bruker ECX 400 spectrometer using CD_3_OD or DMSO-*d*_6_ as a solvent. The chemical shifts were calibrated against residual solvent peaks as the internal standard. The nanogel size and polydispersity (PDI) were measured by dynamic light scattering (DLS) at 25 °C using Anton Paar Litesizer 500 (Austria) equipped with a 658 nm He-Ne laser using back-scattering detection. Transmission electron microscopy (TEM) was performed using a FEI Philips Tecnai 20 under an acceleration voltage of 80 kV. The TEM samples were prepared by dropping 10 μL of nanogel suspension (1.0 mg mL^-1^) on a copper grid followed by lyophilization.

The drug loading content (DLC) and drug loading efficiency (DLE) were determined by HPLC using a reverse phase HPLC column. The flow rate was set as 1 mL/min and the column temperature was set as 25 °C. The HPLC signal was detected at a wavelength of 230 nm using the mixture of acetonitrile and water containing 0.1% trifluoroacetic acid (v/v, 61/39) as eluents. DLC and DLE were calculated using the following formulas:

DLC (*w.t.*%) = (weight of loaded drug/total weight of polymer and loaded drug) × 100%

DLE (%) = (weight of loaded drug/weight of drug in feed) × 100%

# Cell Line

Multi-drug resistant human breast tumor MCF-7/MDR cells were adopted and cultured in RPMI 1640 media supplemented with 10% fetal bovine serum, 1% L-glutamine, antibiotics penicillin (100 IU/mL) and streptomycin (100 mg/mL).

**Supplementary Figure 1.** The MMP depolarization level of MCF-7/MDR cells after 12 h incubation with PBS. The mitochondrial channel was stained with JC-1.

**Supplementary Figure 2.** Intracellular ROS level in MCF-7/MDR cells receiving PBS for 12 h using a probe of DCFH-DA.

**Supplementary Figure 3.** ATP secretion in MCF-7/MDR cells following 12 h incubation with PBS and various drug-loaded nanogels (n = 3, *P < 0.05, **P < 0.01).
